# GLP-1 and Its Role in Glycogen Production: A Narrative Review

**DOI:** 10.3390/biomedicines13071610

**Published:** 2025-06-30

**Authors:** Joseph Lotosky, Xavier Jean, Anungoo Altankhuyag, Saqib Khan, Ashley Bernotas, Alireza Sharafshah, Kenneth Blum, Alan Posner, Panayotis K. Thanos

**Affiliations:** 1 Behavioral Neuropharmacology and Neuroimaging Laboratory on Addictions, Clinical Research Institute on Addictions, Department of Pharmacology and Toxicology, Jacob School of Medicine and Biomedical Sciences, State University of New York at Buffalo, Buffalo, NY 14068, USA; jwlotosk@buffalo.edu (J.L.); anungooa@buffalo.edu (A.A.);; 2 Department of Surgery, State University of New York at Buffalo, Buffalo, NY 14068, USA; 3 Kaleida Health Weight Management Center, Buffalo, NY 14203, USA; 4 Cellular and Molecular Research Center, School of Medicine, Guilan University of Medical Sciences, Rasht 4144666949, Iran; 5 Division of Addiction Research & Education, Center for Sports, Exercise, and Mental Health, Western University of Health Sciences, Pomona, CA 91766, USA; 6 Department of Molecular Biology, Adelson School of Medicine, Ariel University, Ariel 40700, Israel; 7 Department of Exercise and Nutrition Sciences, State University of New York at Buffalo, Buffalo, NY 14068, USA

**Keywords:** glucagon-like peptide-1 (GLP-1), glucagon-like peptide-1 receptor (GLP-1R), glucagon-like peptide-1 receptor agonist (GLP-1RA), glycogen, glycogenesis, thermogenesis, metabolism

## Abstract

Glucagon-like peptide-1 (GLP-1) has emerged as a pivotal regulator in the management of glucose homeostasis, glycogen metabolism, and energy balance, positioning it as a critical therapeutic target for addressing obesity, metabolic syndrome, and type 2 diabetes mellitus (T2DM). GLP-1 receptor agonists (GLP-1RAs) have shown promise for improving glycemic control and reducing weight through appetite regulation, delayed gastric emptying, and energy expenditure modulation. This narrative review explores the mechanisms of GLP-1-mediated glycogen metabolism and energy expenditure, particularly in key tissues—pancreas, liver, skeletal muscle, and adipose tissue. In the pancreas, GLP-1 enhances insulin secretion and beta-cell function. In the liver, it promotes glycogen synthesis via insulin-dependent and potential insulin-independent pathways, involving protein kinase B (AKT) and AMP-activated protein kinase (AMPK) signaling. Skeletal muscle benefits from GLP-1 through increased glucose uptake, AMPK activation, and mitochondrial function, facilitating glycogen storage. In adipose tissue, GLP-1 stimulates brown adipose tissue (BAT) thermogenesis and energy expenditure, contributing to weight loss. This increase in energy expenditure, along with enhanced glycogen metabolism, is a plausible mechanism for the weight loss observed with GLP-1RAs. Despite these advances, significant knowledge gaps remain, particularly regarding the direct hepatic effects of GLP-1, the extent to which it modulates glycogen metabolism in vivo, and its impact on thermogenesis in humans. Future research focusing on both the tissue-specific actions of GLP-1 and its systemic role in energy homeostasis and metabolic regulation will be essential for optimizing its therapeutic potential.

## 1. Introduction

**Figure 1:**
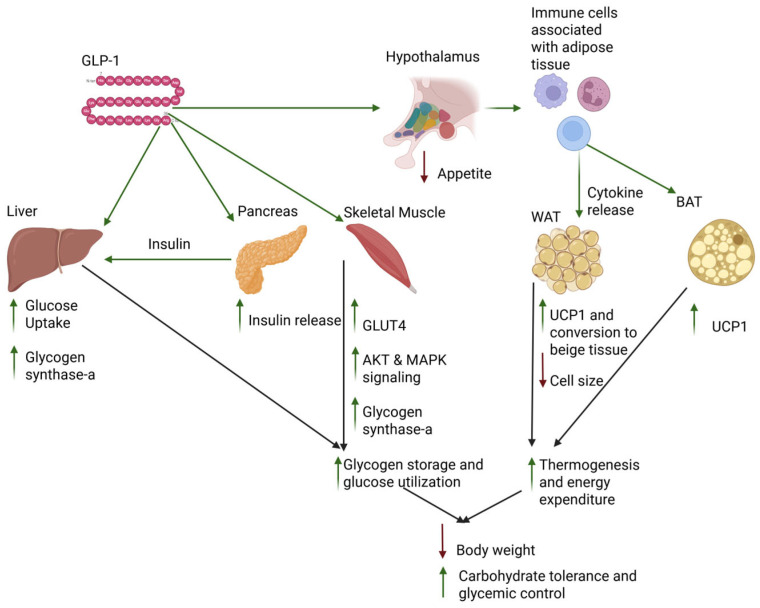
Tissue-specific effects of GLP-1 on glycogen metabolism and thermogenesis. Created in biorender.com.

Glycogen is the main storage form of glucose in the human body. The liver, skeletal muscle, and, to some extent, adipose tissue store glucose as glycogen in the postprandial state [1]. As there is currently a global epidemic of metabolic syndrome, characterized physiologically by a grouping of symptoms such as abdominal obesity, insulin resistance, and hypertension, there is a need for medical and pharmacological interventions to combat this crisis [2]. GLP-1, an incretin hormone that can modulate several metabolic pathways, has become a popular target for pharmacological intervention in metabolic disorders [3]. The efficacy of GLP-1 agonists is primarily attributed to their neurological effects on appetite suppression, food-seeking behavior, and peripheral effects on gastric emptying and insulin secretion [4]. GLP-1 plays a role in glycogen metabolomics and energy expenditure; however, it is not fully understood [5]. Here, we present a review of the current literature surrounding the mechanisms of GLP-1 activity on glycogen metabolism, energy expenditure, and the relevant systems that interplay in inducing these states that can potentially explain the decreases in body weight seen in patients taking GLP-1 agonists, with effects summarized in Figure 1 [6].

GLP-1 is a neuroendocrine hormone synthesized and released by intestinal L-cells in response to a meal, primarily to slow gastric emptying and augment insulin release [7]. Animal models lacking the GLP-1 receptor (GLP-1R) have higher fasting plasma glucose and a decreased ability to clear glucose in response to a meal [8]. Human subjects with impaired glucose tolerance demonstrate a decreased ability to secrete insulin in response to GLP-1 administration [9]. These results highlight that pancreatic beta-cell dysfunction with respect to GLP-1 can exacerbate glucose intolerance and play a role in the progression of chronic conditions associated with impairments in glucose homeostasis, such as type 2 diabetes mellitus(T2DM).

This narrative review utilized the PubMed database to identify relevant manuscripts matching our search criteria and focus of review. The focus of this review was to report on the effects of GLP-1 signaling on glycogen metabolism and total body energy expenditure as it relates to weight management. A total of 19 studies were found to be directly relevant to the scope of this review and were subsequently included with the primary outcome measurements listed (Table 1, Table 2, Table 3 and Table 4). Specific keywords used in this search included “GLP-1, GLP-1R, GLP-1RA, glycogen synthesis, energy metabolism, thermogenesis, liver, pancreas, skeletal muscle, adipose tissue, and BAT.” Other searches were also conducted for foundational and background knowledge for topics such as disease states, treatments, and physiological processes relevant to this review and included terms such as “type 2 diabetes mellitus, obesity, glycogen, glycogenesis, energy balance, metabolism, PASK, TGR5, microbiome, and GLP-1RA”.

## 2. Clinical Disorders and Physiological Pathways Impacted by GLP-1

GLP-1 has emerged as a prominent therapeutic target for type 2 diabetes mellitus (T2DM) and obesity and has recently been shown to have potential therapeutic effects on comorbidities in these populations, such as autoimmune processes, chronic kidney disease (CKD), cardiovascular disease, and Alzheimer’s disease [10]. Autoimmune diseases can develop in the context of chronic, systemic inflammation, where preclinical models of GLP-1 on immune function show promise in directly decreasing the innate immune system production and release of inflammatory cytokines [11]. Kidney disease secondary to T2DM and obesity demonstrates similar pathogenesis, with long-term decreases in glomerular filtration rate (GFR), nephrosclerosis, and progression to end-stage renal disease, where GLP-1 receptor agonist (GLP-1RA) treatment slows CKD progression, measured by an attenuated decline in GFR in patients with either T2DM or obesity and CKD [12,13]. GLP-1RAs have been shown to clinically decrease major cardiac adverse events in patients with T2DM, likely due to a combination of indirect effects of GLP-1 on glycemic control decreasing systemic inflammation and direct effects on endothelial cell function [14]. Neuroinflammation has been shown to exacerbate the onset and progression of Alzheimer’s disease, and preclinical models demonstrate multiple direct effects of GLP-1 on attenuating neuroinflammation in the context of models of Alzheimer’s disease [15]. Clinical studies in patients with T2DM taking the GLP-1RA semaglutide have demonstrated a significantly decreased risk of a first Alzheimer’s disease diagnosis [16]. As this review explores the glycogenic and thermogenic effects of GLP-1, the remainder of this section will focus on T2DM and obesity, two disease states directly impacted by glycogenesis and thermogenesis.

### 2.1. T2DM and Obesity

#### 2.1.1. Introduction to T2DM and Obesity

T2DM is a slow-developing disease that arises from years of insulin resistance and a progressive decline in beta-cell function. Early in the disease, beta cells compensate by increasing insulin production, preventing hyperglycemia. However, as the disease progresses, adipose-driven impairment of beta cells worsens, leading to T2DM when approximately 40–60% of beta-cell mass is lost [17]. The pathogenesis of T2DM can be explained by the twin cycle hypothesis. The twin cycle hypothesis suggests that long-term excess calorie intake leads to triglyceride accumulation in the liver, impairing insulin function. This hepatic insulin resistance increases gluconeogenesis, leading to elevated glucose and insulin levels [17]. Beta-cell dysfunction arises from interactions between environmental factors and molecular pathways. Nearly 90% of individuals diagnosed with T2DM have obesity or are overweight, as classified by a body mass index (BMI) greater than or equal to 25 for overweight and 30 for obesity [18]. Obesity has been previously connected to a concept known as reward deficiency syndrome, where individuals with obesity have intrinsically decreased activity of the brain’s reward circuitry, leading to hyperphagia of highly palatable foods that contribute to significant increases in energy intake [19]. Obesity-related hyperglycemia and hyperlipidemia lead to insulin resistance and chronic inflammation, exposing beta cells to stresses such as inflammation, endoplasmic reticulum stress, oxidative stress, and amyloid stress [20]. Oxidative stress arises due to the excessive production of free radicals, especially reactive oxygen species that severely impact the neutralizing capacity of intracellular antioxidants. Oxidative stress applies its destructive effects by causing damage to deoxyribonucleic acid (DNA), proteins, and lipids [21]. In individuals with obesity, adipose tissue becomes a source of inflammation, producing higher levels of proinflammatory markers like tumor necrosis factor-alpha (TNF-α), interleukin-6 (IL-6), and monocyte chemotactic protein-1 [22]. This inflammatory state contributes to insulin resistance and provokes beta-cell dysfunction in the pancreas. An important marker of adipose tissue health is plasma adiponectin, which is associated with insulin sensitivity. Adiponectin has a variety of functions, including anti-inflammatory, anti-fibrotic, and insulin-sensitizing effects [23]. Adiponectin levels are low in obesity. In the liver, adiponectin decreases the influx of fatty acids and increases fatty acid oxidation. Adiponectin serves as a reliable marker for insulin sensitivity, with its levels inversely correlating with the degree of insulin resistance and metabolic dysfunction. The size of beta cells is imperative in understanding T2DM in addition to its function. Normally, beta-cell mass is maintained through a balance between neogenesis, replication, hypertrophy, and apoptosis [24]. However, in those who have obesity or insulin resistance, increased insulin demand leads to an expansion in the number and size of islets and beta cells [24]. In those who are diagnosed with obesity and/or T2DM, there is increased apoptosis and decreased neogenesis, which causes a reduction in beta cell mass.

In T2DM patients, insulin cannot regulate hepatic glycogen synthesis or glucose production, and increased hepatic gluconeogenesis is the primary cause of fasting hyperglycemia in T2DM [25]. When skeletal muscle develops insulin resistance, excess glucose in the form of diacylglycerol enters the liver and activates protein kinase C epsilon [26]. This protein results in a decrease in proximal insulin signaling and prevents excess glucose from entering the hepatocytes. T2DM patients also see an abundance of free FAs in the plasma, which reduces insulin-regulated glucose metabolism [27]. Serine/threonine kinase AKT prompts insulin effects on the liver, such as glycogen synthesis and the suppression of hepatic glucose production. Akt signaling plays a vital role in activating GLUT-4, which moves to the cell surface and transports glucose into the cell [28]. Ceramide blocks the AKT pathway, which causes impairment of glycogen synthesis, glucose transport, and the inhibition of insulin receptors and signaling [29]. Specifically, ceramide inhibits respiratory chain complexes I and III, increasing mitochondrial membrane permeability and forming channels for ion and protein release. This disruption impairs mitochondrial function, leading to reduced glucose and lipid utilization [29]. Insulin resistance contributes to the downregulation of muscle contractile protein synthesis by inhibiting insulin’s anabolic signaling pathway and simultaneously upregulating protein degradation via the ubiquitin–proteosome pathway [30]. Along with the debilitation of skeletal muscle, as individuals with T2DM age, there is a gradual decline in mitochondrial function in human skeletal muscle along with a decrease in muscle mass, strength, and overall muscle function [31].

#### 2.1.2. Current Pharmacological Treatments for Obesity and T2DM and Their Mechanism of Action

Obesity has a significant role in negative health outcomes due to an increased risk of many pathologic states, including cardiovascular disease, metabolic dysfunction, and cancer [32]. Historically, adverse effects of pharmacologic interventions for obesity have generated concern over the use of pharmaceuticals [33]. Newer approaches to medical weight loss have focused on minimizing side effects [34]. Currently, six drugs are approved by the FDA for long-term use for patients diagnosed with obesity [35]. Orlistat, a pancreatic and gastric lipase inhibitor, inhibits the breakdown of ingested triglycerides, reducing the caloric load and leading to modest weight loss. Phentermine-topiramate exerts its anti-obesogenic effects by suppressing appetite through a catecholamine-mediated mechanism of phentermine. The exact mechanism of topiramate is not well understood, but it augments weight loss induced by phentermine through suspected modulation of GABA receptors, carbonic anhydrase inhibition, and glutamate antagonism [36]. The combination drug bupropion/naltrexone activates pro-opiomelanocortin (POMC) neurons, releasing alpha-melanocyte-stimulating hormone, providing positive effects on body weight regulation [37]. The following class of medications involves GLP-1R activity, the focus of this review. Liraglutide, the first GLP-1RA FDA-approved medication, induces weight loss through GLP-1R activity. Semaglutide, the next FDA-approved GLP-1RA medication, demonstrates greater weight loss in patients with obesity, mediated by its increased half-life compared to liraglutide through modification to inhibit degradation by dipeptidyl-peptidase-4 (DPP-4) [38]. The most recently approved FDA medication for the treatment of obesity, tirzepatide, exerts its physiologic effects as a dual GLP-1R and Gastric Inhibitory Polypeptide Receptor (GIP-R) agonist with better efficacy than the standalone GIP-R agonist semaglutide [39].

#### 2.1.3. Current Pharmacological GLP-1RAs with a Comparison of Their Efficacy Pharmacokinetics/Dynamics and Other Relevant Comparisons

Many GLP-1RAs have shown efficacy in improving glycemic control and decreasing body weight compared to placebo [40]. Currently, there are eight FDA-approved GLP-1RA drugs for the treatment of T2DM and two for the treatment of obesity [41]. Depending on the formulation and half-life of the GLP-1RA, the physiological effects may differ. Where short-term acting GLP-1RAs primarily exert their effects by modulating and delaying gastric emptying and subsequent changes in postprandial glucose uptake, longer-acting GLP-1RAs affect both basal and postprandial glucose regulation [42]. The LEAD-6 trial, a 26-week-long study comparing a short-acting GLP-1RA, exenatide, to a longer-acting GLP-1RA, liraglutide, demonstrated glycemic control improvements in both study groups, with a larger reduction in Hemoglobin A1c (HbA1c) in the liraglutide group [43]. A subsequent trial, the DURATION-6 trial, compared the effects of a once-weekly dose of exenatide against a daily dose of liraglutide in study groups and found improvements in glycemic control in both groups, with again greater reductions in HbA1c in the liraglutide group [44]. In the SUSTAIN-3 trial, the effects of the GLP-1RA semaglutide were compared to that of exenatide extended-release, where the semaglutide group had significantly greater improvements in glycemic control than the exenatide extended-release group [45]. More recently, the SUSTAIN-10 trial compared the effects of semaglutide against that of liraglutide in glycemic control, revealing a greater effect of reducing HbA1c and weight in the semaglutide group, yet gastric side effects were more common in this group [46]. A meta-analysis of the effects of the current GLP-1RAs on body weight, glycemic control, and lipid profile revealed that tirzepatide, a dual GLP-1RA and GIP-RA, has the highest efficacy in improving glycemic control compared to other GLP-1RAs, suggesting a beneficial effect of targeting the GIP-R along with the GLP-1R. In a mouse model of the GLP-1R agonist semaglutide, researchers found that semaglutide administration led to a significant decrease in body weight in treated mice while attenuating the expected decrease in energy expenditure due to the decrease in weight [47]. Current research on the effects of GLP-1 and its respective analogs on energy expenditure in humans is inconclusive at this time, but at least one study has demonstrated an increase in energy expenditure [48]. Another human study demonstrated a slight decrease in energy expenditure, but this decrease is not proportional to the weight loss of the experimental group receiving GLP-1 agonists, suggesting that GLP-1 agonists can attenuate some of the decrease in energy expenditure expected with a decrease in weight [49]. This study also demonstrated that the GLP-1R agonist liraglutide caused a shift in energy metabolism from carbohydrates to lipids, which could potentially be explained by GLP-1R activation inducing BAT thermogenesis and increasing hepatocyte and skeletal muscle cell glycogenesis, sparing glycogen. Although there is debate as to whether current GLP-1 agonists can readily cross the blood–brain barrier, there is evidence to suggest that most do and exhibit CNS activity in humans [50].

### 2.2. Blood Glucose Homeostasis and Hepatic Glycogen Metabolism

Blood glucose levels are meticulously maintained within a functional range through interactions between several organ systems to ensure that metabolic demands are met without adverse toxic effects. Disruption of this system is linked to disease states, especially T2DM, and obesity [51]. In the fed state, this system involves, but is not limited to, (A) glucose absorption through gut endothelial cells, (B) insulin release from pancreatic beta cells, (C) hepatic glucose uptake, (D) muscle cell insulin-mediated glucose uptake, (E) adipocyte cell insulin-mediated glucose uptake, and (F) the hypothalamic regions modulating the peripheral insulin response through neuropeptide and neurotransmitter signaling [52]. The net effect of this system is to store excess circulating glucose as glycogen primarily in the liver and skeletal muscle, convert excess glucose to lipids in adipocytes, and provide sufficient glucose to tissues for energy production [53].

The liver is responsible for maintaining circulating blood glucose levels through the degradation of glycogen during a fasting state and storing glucose as glycogen in the fed state [54]. In the fed state, hepatic tissue responds to circulating insulin by activating AKT, which inactivates glycogen synthase kinase-3 (GSK-3), preventing its inhibition of glycogen synthase. Glycogen synthase is then active and can catalyze glycogen synthesis [55]. In the fasted state, hepatic tissue responds to circulating glucagon and catecholamine levels to induce PKA activation of glycogen phosphorylase, the enzyme responsible for glycogen catabolism [56]. As glycogen is catabolized to glucose-6-phosphate, it can further be metabolized to glucose for transport into the peripheral circulation to maintain basal glucose concentrations in a fasted state or when energy demands increase. While this explanation is an oversimplification of the process, a comprehensive review is found elsewhere [56]. A basal-level explanation of the antagonistic processes of glycogen metabolism in hepatic tissue was provided to ensure adequate context to understand GLP-1-mediated effects in the liver. Data from 25 healthy volunteers displayed that free fatty acid metabolism is more sensitive to insulin than glucose metabolism, suggesting cooperation between glucose and free fatty acids in energy homeostasis, with free fatty acids playing the role of a buffer to support glucose stability [57].

### 2.3. Energy Balance and Thermogenesis

The complex interplay between internal signals, neural circuits, energy absorption, and energy utilization results in energy balance homeostasis [58]. A disruption to this fine-tuned physiological process, where net energy consumption outweighs that of energy utilization, can lead to metabolic dysfunction. This metabolic dysfunction in turn can result in obesity [59]. Many hormones, cortical areas, and metabolic regulators are implicated in energy balance, such as leptin, adiponectin, and GLP-1 [60]. Recently, attention has been directed to the role of non-exercise activity thermogenesis (NEAT) as it relates to human energy homeostasis [61]. A component of NEAT, brown or beige adipose tissue (BAT)-induced thermogenesis, occurs independent of movement through the mitochondrial protein that decouples oxidative phosphorylation through the mitochondrial membrane protein uncoupling protein 1 (UCP-1) [62]. BAT has recently been implicated as a potential target of obesity and metabolic disorders, where induction of UCP-1-mediated NEAT thermogenesis may be a potential therapeutic target [63]. Current research highlights alternative cellular systems involved in NEAT-mediated thermogenesis, such as an adipocyte futile cycle in fatty acid breakdown and subsequent synthesis; catecholamine- and adrenergic-receptor-dependent thermogenesis; and creatine cycling as examples [64]. GLP-1 activity has recently been implicated in increasing BAT thermogenesis, and a further exploration of this interaction may better explain the full physiologic effects of GLP-1 agonists as they contribute to weight loss [65].

## 3. GLP-1 and Its Role in Glycogen Metabolism and Energy Balance

### 3.1. Introduction to GLP-1 Physiology

The primary, most well-studied mechanism of GLP-1 activity on glycogen synthesis lies in its incretin abilities to increase pancreatic beta-cell insulin secretion in postprandial states [66]. GLP-1R activation in pancreatic beta cells increases the insulin response to circulating glucose, leading to an immediately recognized glucose transporter-4 (GLUT-4)-mediated glucose uptake and storage as glycogen in adipocytes and myocytes [67]. In all glycogen-containing cell lines, high blood glucose levels increase the activity of phosphoglucose mutase, which catalyzes the reaction of glucose 6-phosphate (G6P) to glucose 1-phosphate (G1P). G1P is then converted to UDP-glucose through the enzyme UDP-glucose pyrophosphorylase (UGPase), where UDP-glucose acts as the monomeric substrate in the anabolic process of glycogen synthesis, mediated by the enzyme glycogen synthase. GLP-1 regulation of systemic glycogen metabolism and glucose utilization is complex and is also mediated through central nervous system structures and neural circuits [68]. Exploring the tissue-specific effects of GLP-1 activity in depth will elicit a better understanding of the gross effects of GLP-1 activity on this phenomenon.

### 3.2. Tissue-Specific Effects of GLP-1 Activity

#### 3.2.1. GLP-1 Effects on the Pancreas

**Figure 2:**
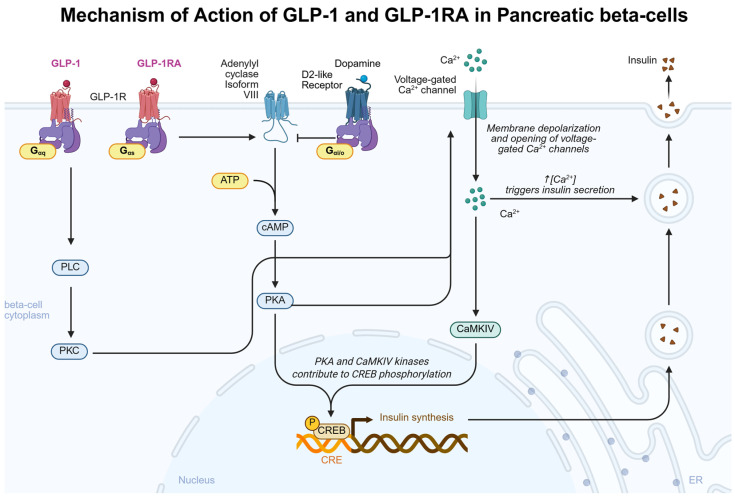
Mechanism of action of GLP-1 and GLP-1RA in pancreatic beta cells. Physiological GLP-1 exists in picomolar concentrations and binds to GLP-1R coupled to G-αq, exerting physiologic effects through PLC/PKC and activation of voltage-gated calcium channels. Supraphysiological concentrations of GLP-1 and GLP-1RA will also bind to GLP-1R coupled to G-αs, exerting physiological effects through cAMP/PKA signaling, increasing insulin gene expression, and activating voltage-gated calcium channels. Created in biorender.com.

GLP-1 acts as an insulinotropic hormone, increasing pancreatic islet cell insulin mRNA levels and insulin biosynthesis in response to GLP-1R stimulation [69] [Figure 2]. Once activated, the GLP-1R G-αs protein activates adenylate cyclase isoform VIII, the converging adenylate cyclase isoform that is responsible for the interaction between glucose and GLP-1 in regulating insulin levels [70]. Downstream of adenylate cyclase VIII and resultant increases in cyclic adenosine monophosphate (cAMP), GLP-1 increases activation of protein kinase A [71]. Increases in cAMP are cell-specific, where GLP-1 failed to induce any detectable changes in alpha cells at a concentration of 1 nmol/L but was able to cause a dose-dependent increase in cAMP in beta cells, with a significant physiologic effect beginning at 10 pmol/L [72]. These results align with physiologic GLP-1 concentrations, where basal GLP-1 levels range from 5 to 15 pmol/L [73]. However, as maximal GLP-1-stimulated cAMP increases occur in the nanomolar concentration, research has elucidated alternative signaling pathways involved in GLP-1-mediated insulin secretion, particularly that of the protein kinase C (PKC) [74]. Picomolar concentrations of GLP-1 activate PKC and increase insulin secretion in mouse islet cells independent of protein kinase A (PKA) activation [75]. The pharmacologic GLP-1RA semaglutide has been shown to bind GLP-1R with higher affinity than GLP-1 [76] and achieve nanomolar concentrations with pharmacologic interventions [77], suggesting that exogenous GLP-1R therapy may induce PKA-associated insulin secretion in pancreatic tissue that is not achieved with physiologic GLP-1 activity. A summary of the results of pre-clinical effects of GLP-1 on pancreatic beta-cells is presented in Table 1. It should be mentioned that dopamine acts as a negative regulator of insulin release at the level of dopamine cells, where dopamine acts at beta-cell D2-like dopamine receptors to inhibit insulin secretion [78]. Therefore, when discerning the effects of GLP-1 on insulin release, it may be important to understand peripheral dopamine and how it could influence this pathway. Further research is needed to determine if differential beta-cell pancreatic signaling pathways are activated through exogenous GLP-1R therapy and endogenous GLP-1 signaling.

**Table 1:** Preclinical studies of GLP-1-mediated effects on pancreatic tissue.

GLP-1 Intervention	Species	Sex	Regimen	Dose	Administration Route	Outcomes	Reference
GLP-1 (7–37)	Rat pancreatic islet cells	N/A	1-day cell culture incubation	0.5 µM	N/A	↑ cAMP ↑ Insulin mRNA ↑ Insulin release	[69]
GLP-1	Rat pancreatic beta cells	Male	15 min cell culture incubation	10 nM	N/A	↑ cAMP ↑ Type VIII adenylate cyclase	[70]
GLP-1	Mice pancreatic islet and beta cells	Female	N/A	1 µM	N/A	↑ Insulin ↑ Ca^2+^ channel activity	[75]

#### 3.2.2. GLP-1 Effects on the Liver

As endogenous intestinal-secreted GLP-1 is absorbed into the liver through the portal vein, the liver is highly involved in GLP-1 regulation [73]. The liver is the organ responsible for maintaining circulating glucose homeostasis through glucose and glycogen anabolism and catabolism [56]. There has been a debate whether hepatocytes can directly respond to GLP-1 or if the hepatic response to GLP-1 is mediated by pancreatic beta-cell GLP-1 activity [79]. GLP-1 administration significantly increases glucose uptake in the liver and attenuates glucose-administered hyperglycemia in a dose- and rate-dependent manner [80]. In a canine model, GLP-1 was found to be sufficient in inducing hepatic glucose disposal when delivered in the portal system, but not when delivered into the peripheral circulation [81]. More recent research suggests that GLP-1R is localized within hepatocytes and that GLP-1R activation results in the cellular internalization of this receptor [82]. Conversely, GLP-1R was not found in primate liver tissue with no direct evaluation of human liver tissue GLP-1R distribution [83]. Further research is needed to more clearly elucidate both if GLP-1-mediated glucose uptake in hepatic tissue is exclusively through extrahepatic GLP-1R activation and if GLP-1R is distributed in human hepatic tissue.

GLP-1 activity plays a foundational role in hepatocyte glycogen synthesis by increasing dose-dependent glycogen synthase-a activity [84]. GLP-1-deficient mice demonstrate decreased liver glycogen accumulation downstream of AKT and glycogen synthase a signaling [85]. Although glycogen synthesis and intracellular glycogen levels proportionally inhibit AMPK activity [86], the increase in glycogen seen in patients receiving GLP-1 agonists may be partially due to GLP-1R/AMPK activation that overrides basal glycogen inhibition of AMPK. GLP-1 has been shown to have insulin-independent effects on hepatic glucose uptake [87]. The insulin-independent effects on glycogen synthesis are mediated through GLP-1 signaling through phosphoinositide 3-kinase (PI3K), AKT, PKC, and protein phosphatase-1 (PP-1) activity, but the interplay between these enzymes is not currently fully understood in GLP-1-mediated glycogen metabolism [88].

Mice with mutations disrupting AMPK binding to glycogen in the liver and skeletal muscle showed increased adiposity and AMPK instability [89]. Mice with disrupted AMPK-glycogen binding displayed glucose intolerance associated with reduced fat oxidation and reductions in AMPK protein content in comparison to WT mice. These mice had reduced liver glycogen in the fed state and no repletion of skeletal muscle glycogen in response to fasting and refeeding. In a separate insulin-clamp experiment, GLP-1R-deficient mice demonstrated decreased liver glycogen content, increased muscle glycogen content, and hyperglycemia during exercise compared to healthy controls, demonstrating insulin-independent effects of GLP-1 on glycolytic flux [90]. These results potentially demonstrate a role of GLP-1 in preferentially acting on the liver and inducing hepatic glucose disposal and glycogenesis over that of skeletal muscle, given that endogenous GLP-1 is normally secreted in response to nutrient absorption following a fed state.

To further understand the effects of PI3K/AKT signaling in glycogen metabolism, an investigation of non-GLP-1 compounds in this phenomenon was conducted. Dichloroacetate, a by-product of water chlorination, was found to increase glycogenesis through a PI3K/AKT mechanism independent of insulin signaling [91], demonstrating another bioactive molecule that has insulin-independent effects on glycogen synthesis through a common mechanism. Glucocorticoid receptor beta (GRb) has also been found to increase glycogen synthesis through the PI3K/AKT pathway independent of insulin signaling, highlighting the importance of this secondary messenger signaling in insulin-independent mechanisms underlying glycogen synthesis [92]. GLP-1 has been found to antagonize glucagon-induced glycogenolysis in hepatocytes [93], providing another plausible mechanistic interaction by which GLP-1 could increase hepatocyte glycogen stores. A summary of preclinical effects of GLP-1 on hepatic tissue is presented in Table 2. A greater understanding of the mechanisms of GLP-1-induced glycogen metabolism in hepatocytes could provide clinical insight and guide future research. Decreased hepatic glycogen content has been shown in preclinical models to contribute to insulin resistance and hepatic steatosis, two physiologic states directly implicated in diseases of metabolic dysfunction such as T2DM and obesity [94]. Hepatic glycogen content has also been shown to attenuate hepatocyte triglyceride accumulation, which can directly influence systemic metabolic health and potentially provide new mechanistic pathway targets for metabolic dysfunction and disease [95].

**Table 2:** Preclinical studies of GLP-1-mediated effects on liver tissue.

GLP-1 Intervention	Species	Sex	Regimen	Dose	Administration Route	Outcomes	Reference
GLP-1	Rat liver cells	Male	N/A	1nM	GLP-1 added to cell cultures	↑ GS-a	[84]
GLP-1R homozygous knockout	Mice	Male	N/A	N/A	N/A	↓ Hepatic glycogen content	[85]
GLP-1	Mongrel Dogs	Both	Continuous during experiment	10 and 20 pmol·kg^−1^·min^−1^	GLP-1 intraportal infusion	↑ Hepatic glucose uptake	[80]
GLP-1	Mongrel Dogs	Both	Continuous during experiment	7.5 pmol·kg^−1^·min^−1^	GLP-1-(7–36) peripheral vein infusion	↑ Hepatic glucose uptake independent of insulin secretion	[87]
GLP-1	PVH zone rat liver cells	Male	Culture incubation	1000 nM	GLP-1	Inhibition of 0.1 nM glucagon-induced glycogenolysis	[93]

#### 3.2.3. GLP-1 Effects on Skeletal Muscle

**Figure 3:**
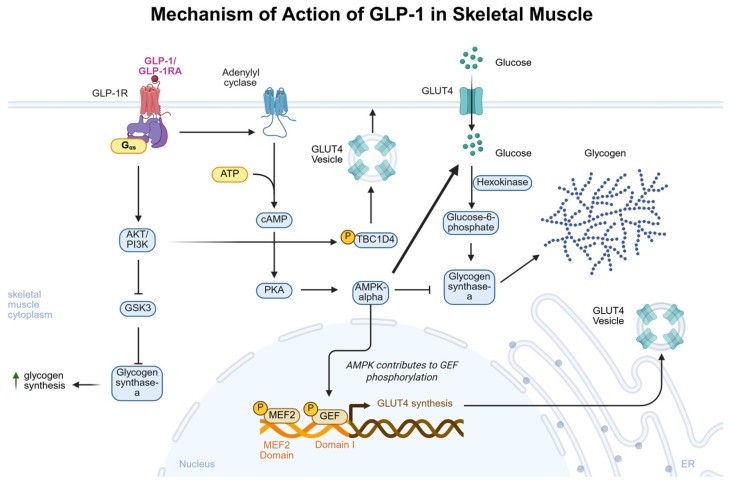
Mechanism of action of GLP-1 in skeletal muscle. GLP-1/GLP-1RA binds to GLP-1R coupled to G-αs and exerts physiological effects through both AKT/PI3K and cAMP/PKA/AMPK-α signaling pathways. Increased AKT/PI3K signaling primarily increases glycogen synthesis by inhibiting GSK3 and increasing GLUT4 vesicle translocation to the cell membrane by phosphorylation of TBC1D4. The AKT/PI3K pathway is disrupted in T2DM and may partially explain decreased effects of GLP-1RA in T2DM. Increased cAMP/PKA/AMPK-α signaling increases GLUT4 gene expression through GEF phosphorylation and directly inhibits glycogen synthase-alpha. However, increased GLUT4 expression increases intracellular glucose uptake and glucose-6-phosphate production, a positive allosteric modulator of glycogen synthase-alpha, where current research suggests this effect leads to a net increase in glycogen synthase-alpha activity and subsequent glycogen synthesis through AMPK-α signaling. Created in biorender.com.

Increasing GLP-1 activity, through endogenous or exogenous GLP-1 or GLP-1R agonists, increases glycogen synthesis and glucose uptake in skeletal muscle via AMP-activated protein kinase (AMPK) activation [96]. AMPK activation has been shown to increase GLUT4 enhancer factor (GEF) phosphorylation, a transcription factor that interacts with myocyte enhancer factor 2 (MEF2) to induce GLUT4 gene expression [97]. Mice with mutations disrupting AMPK binding to glycogen in skeletal muscle showed increased adiposity and AMPK instability, where glycogen binding is essential for AMPK stability and energy homeostasis [98]. In cultured human myocytes, GLP-1 mediated an increase in glycogen synthase-a activity, where exendin-4 led to dose-dependent increases in both glucose uptake and glycogen synthase-a [99]. In skeletal muscle, upstream of glycogen storage, the AKT/PI3K has been necessarily implicated in glycogen synthesis [51]. In patients with T2DM, the AKT/PI3K signaling pathway is dysfunctional, contributing to the pathophysiology of this disease [100]. PI3K induction by GLP-1 agonists, independent of insulin activity, potentially increases GLUT-4 protein levels [101]. In a mouse model of T2DM, GLP-1 and exendin-4 were unable to stimulate skeletal muscle glucose uptake and glycogen storage due to the reduced expression of AKT in these cells [102]. When AKT is inhibited, skeletal muscle glucose uptake and glycogen synthesis are impaired [103], implying that robust AKT signaling is necessary for GLP-1-dependent increases in skeletal muscle glycogen synthesis. PI3K-deficient mice demonstrate muscle insulin resistance, increased adiposity, and hyperlipidemia, further supporting that the AKT/PI3K pathway regulates skeletal glycogen synthesis and metabolic health [104]. A study of secondary messenger inhibitors during GLP-1 or insulin administration revealed that AKT/PI3K and mitogen-activated protein kinase (MAPK) signaling are necessary for GLP-1 to exert its glycogenic effects [105]. In human myocytes from patients with obesity, GLP-1 alleviated obesity-induced reductions in muscle glucose uptake, but not in T2DM or healthy subject myocytes [106]. These results demonstrate that GLP-1 may preferentially alleviate dysregulated glucose uptake and glycogen metabolism in skeletal muscle in the context of obesity by rescuing deficits in AKT/PI3K signaling, but not in conditions of T2DM or normal physiology. The mechanisms of action of GLP-1 in skeletal muscle tissue are summarized in Figure 3. A summary of the results of preclinical trials of GLP-1 on skeletal muscle are presented in Table 3. Future research exploring the physiology of GLP-1-induced skeletal muscle AMPK and AKT/PI3K interactions will allow researchers and clinicians to better understand individual differences in GLP-1 responses based on their baseline skeletal muscle metabolic health.

The effects of GLP-1 on skeletal muscle metabolism may also be attributed to increased oxidative capacity, mitochondrial biogenesis, and increased lipid utilization [107]. A shift in energy metabolism towards lipid utilization is glycogen sparing, which would at least partially explain increases in skeletal muscle glycogen content due to a decrease in glycogen catabolism. As both the liver and skeletal muscle store most of the glycogen in humans, current research supporting increased glucose uptake and glycogen synthesis within these tissues provides plausibility in explaining the phenomenon where individuals prescribed GLP-1 agonists can consume greater amounts of carbohydrates while still losing weight.

**Table 3:** Preclinical studies of GLP-1-mediated effects on muscle tissue.

GLP-1 Intervention	Species	Sex	Regimen	Dose	Administration Route	Outcomes	Reference
Exendin-4	C2C12 mouse skeletal myoblast cells	N/A	5 days	0.5 μM	N/A	↑ glycogen ↑ membrane Glut-4	[96]
GLP-1 overexpression	Mice	Male	N/A	N/A	GLP-1-AAV	↑ gene expression of chemokine, AMPK, PI3K/AKT, PLD, cAMP ↑ pAMPK	[96]
GLP-1	Skeletal muscle satellite cells	Male	Cell culture incubation	100 nM	N/A	↑ PI3K/AKT	[101]
Exendin-4	Streptozotocin (STZ)-induced rats with diabetes	Male	Culture incubation	10^−9^ M Ex-4	N/A	↑ PI3K, p70s6K, MAPKs phosphorylation No effect on AKT phosphorylation or glycogen synthase	[102]
GLP-1	Rat skeletal muscle strips	N/A	10 min culture incubation	10^−10^ M	N/A	↑ PI3K/AKT, p70s6K, p44/42 ↑ glycogen synthase-a activity	[105]
GLP-1	Cultured myocytes from humans with obesity	N/A	3 min culture incubation	10^−9^ M	N/A	↑ AKT, p70s6K, p44/42 phosphorylation ↑ glycogen synthase activity	[106]

#### 3.2.4. GLP-1 Effects on Adipose Tissue

Current research also hypothesizes that GLP-1 and its pharmacologic agonists increase energy expenditure. GLP-1 may increase energy expenditure by activating brown adipose tissue (BAT) thermogenesis through sympathetic nervous system activity induced by GLP-1 activity in the central nervous system (CNS) [108]. A loss of neuronal GLP-1R decreases energy expenditure and uncoupling protein 1 expression (UCP1), where GLP-1 activity is necessary for the dorsomedial hypothalamus to activate brown-adipose-tissue thermogenesis [109]. In the hypothalamic ventromedial nucleus, GLP-1 acts to increase BAT thermogenesis independent of nutrient intake [110]. Further research implies immune activity, where invariant natural killer T-cells (iNKTs) induce BAT thermogenesis through a GLP-1 mechanism [111]. Weight loss is less in rodent models where iNKTs are deficient, even when GLP-1R agonists still induce satiety, implicating GLP-1 in increasing energy expenditure [111]. GLP-1 stimulation of adipose tissue monocytes increases IL-6 signaling in adipocytes, leading to increased UCP-1 expression and glucose metabolism [112].

Adipocyte glycogen synthesis and metabolism are necessary for BAT thermogenesis induction [113], providing another plausible mechanism by which the effects of GLP-1 converge on increasing carbohydrate consumption while simultaneously leading to increased energy expenditure and subsequent weight loss. Deletion of the glycogen synthase-1 gene in mouse adipocytes attenuated glycogen synthesis and UCP-1 upregulation under conditions that normally stimulate UCP-1 activity, such as cold exposure or B3-adrenergic receptor activation [114]. Together, these results demonstrate that GLP-1 stimulation of adipose tissue from neural and immunologic cell lines increases thermogenesis. A summary of pre-clinical results of GLP-1 effects on adipose tissue is presented in Table 4. This pathway is influenced by peripheral dopamine activity, as coincubation of dopamine, a GLP-1RA, and a dopamine D2 receptor antagonist increases AMPK phosphorylation and subsequent activation of thermogenesis, likely through a dopamine D1-receptor-mediated mechanism [115]. If patients prescribed GLP-1R agonists can consume higher levels of carbohydrates and energy while still losing weight, a combination of increased glycogen production and energy metabolism may lead to this phenomenon. Highlighting the implication of the effects of GLP-1 in adipose tissue, dopamine D1 and D4 receptor expression was decreased and correlated with GLP-1R expression in adipose tissue from patients with obesity compared to healthy controls, where targeting this pathway may have future clinical relevance [115]. Further investigation into the possible effect of GLP-1-mediated effects on adipocyte glycogen synthesis may be beneficial in better understanding the role of GLP-1 in mediating adipocyte increases in energy metabolism.

**Table 4:** Preclinical studies of GLP-1-mediated effects on adipose tissue.

GLP-1 Intervention	Species	Sex	Regimen	Dose	Administration Route	Outcomes	Reference
GLP-1	Mice	Male	4 days	0.75 nmol/day	Intracerebroventricular injection	↑ iBAT thermogenesis	[108]
GLP-1	Rats	Male	Single dose	0.5 μg	Dorsomedial hypothalamic injection	↑ iBAT thermogenesis	[109]
Liraglutide	Mice	Male	Single dose	3 μg	Intracerebroventricular (ICV)	↑ iBAT thermogenesis ↑ UCP1, UCP3, ADRB1, FGF21, PRDM16 ↑ White adipose tissue UCP-1 and PRDM16	[110]
Liraglutide	Mice	Male	5 days following 6–8 weeks of high-fat diet	50 μg/kg	Intraperitoneally	↑ iNKT cell count and IL-10 production ↑ White adipose tissue UCP-1, FGF21, adiponectin PGC1a, and Cidea	[111]
Liraglutide	Mice	Male	2 and 4 weeks	0.2 mg/kg	Intraperitoneal injection	↑ UCP1 ↑ AMPK phosphorylation ↓ Fat mass	[112]

## 4. Additional Biological Processes Impacted by GLP-1 That Impact Glycogen Metabolism and Energy Homeostasis

### 4.1. GLP-1 Effects on Gastric Emptying

GLP-1 receptor agonists delay gastric emptying, yet this effect lessens with prolonged use [116]. Dual gastric inhibitory polypeptide (GIP) and GLP-1RA tirzepatide were compared to GLP-1 receptor agonists in their effect on gastric emptying in clinical and non-clinical studies. In mice with diet-induced obesity, tirzepatide delayed gastric emptying similarly to semaglutide, but the effect wore off after two weeks of treatment. In T2DM patients, a single dose of tirzepatide delayed gastric emptying with a reduced effect over time. However, T2DM patients using a dose-escalation schedule showed a persistent gastric emptying delay over time. These results indicate that the impact of tirzepatide on gastric emptying is like that of selective GLP-1 receptor agonists [117].

Retained gastric contents during upper gastrointestinal endoscopy are more frequently observed in patients on GLP-1 receptor agonists [118]. A matched pair case-control study found a significantly higher proportion of gastric residue in those treated with GLP-1Ras through esophagogastroduodenoscopy [119]. Another study evaluated the relationship between gastric emptying and GLP-1 secretion in individuals with T2DM by measuring GLP-1 responses to a starch meal and intraduodenal infusions of glucose, fat, and protein. While the GLP-1 response to the starch meal did not correlate with gastric emptying time, significant interindividual variability was observed in GLP-1 responses to intraduodenal nutrients, with the gastric emptying rate partially influenced by GLP-1 responsiveness to glucose [120].

A human study showed that GLP-1 significantly delayed gastric emptying but did not affect gastric muscle contractility or water homeostasis, suggesting GLP-1 inhibits gastric emptying through an indirect mechanism rather than altering muscle contractility directly [121]. In a study of children with obesity compared to healthy peers, there was no significant link between hormone levels, including GLP-1, and gastric motility regardless of obesity or GERD status, suggesting childhood obesity does not yet cause physiological changes in gut motility or GLP-1 signaling effects on gut motility [122].

Patients one year post-esophagectomy showed variable gastric emptying speeds and significantly shorter cecal arrival times. However, these results did not correlate with GLP-1 release, suggesting other factors may drive the GLP-1 response after esophagectomy [123]. Patients post-laparoscopic sleeve gastrectomy (LSG) showed significantly accelerated gastric emptying and elevated GLP-1 plasma levels compared to before the LSG. Higher GLP-1 concentrations were observed as gastric retention decreased. This negative correlation suggests that faster emptying stimulates increased GLP-1 production in the distal bowel [124]. Blocking GLP-1 receptors with exendin-9,39 did not affect caloric intake in post-bariatric surgery patients, but it did reduce caloric intake in their weight-matched control group [125].

### 4.2. GLP-1 Effects on Appetite Regulation

GLP-1R is expressed in the lateral hypothalamus (LH), where animal studies have shown that activation of these receptors decreases appetite [126]. A cadaveric study found a decrease in GLP-1R expression in the LH that correlated with increased BMI, suggesting that reduced GLP-1R signaling in the LH may contribute to dysregulated feeding behavior in individuals with obesity [127]. It may be important to consider the role of dopaminergic signaling in feeding behavior in the context of GLP-1, as individuals with obesity have been found to have decreased D2R expression in striatal regions that project to the LH, suggesting that obesity may lead to decreased satiability and increased hyperphagia through this circuit [128].

Acute administration of the GLP-1RA exenatide resulted in reduced feelings of hunger, which is due to an increase in functional connectivity of the nucleus tractus solitarius with the hypothalamus and thalamus [129]. Pharmacologic GLP-1RA administration has been shown to have various reductions in food consumption scores and an increase in perceived fullness in humans with obesity, where postprandial plasma levels of GLP-1 decreased and peptide YY (PYY) levels increased with the GLP-1RA liraglutide [130]. In another study, a double-blind, parallel-group trial was conducted in 72 adults with obesity, randomized to once-weekly subcutaneous semaglutide or placebo for 20 weeks. This weekly semaglutide suppressed appetite, improved control of eating, and reduced food cravings, ad libitum energy intake, and body weight vs. placebo [131].

In mice, the distribution of GLP-1R-expressing neurons and the axonal pathways of GLP-Q-producing neurons from the nucleus tractus solitarius (NTS) in the mouse brain were analyzed. Using the GLP-QR agonist Exendin-4 (Exn-4) increased firing rates in arcuate nucleus (ARC) GLP-1R and POMC neurons, and the chemogenetic activation of ARC GLP-1R neurons significantly reduced food intake [132]. To further analyze the effects of GLP-1 on appetite, fifty participants were randomized to either lixisenatide or liraglutide for a treatment of 10 weeks. Both GLP-1 RAs reduced macronutrient intake, with liraglutide treatment specifically increasing lipase, while both treatments improved fecal elastase and serum beta-carotene levels [133]. GLP-1 receptor agonists demonstrate efficacy in modulating reward pathway dysfunction by normalizing insulin resistance and stabilizing glycemic variability, thereby reducing cravings and subsequently suppressing appetite [134]. GLP-1, released from intestinal L-cells, activates intestinofugal neurons (IFNs) that project to sympathetic pathways, reducing gastric motility and appetite through the ileal brake reflex [135]. In a 12-week, double-blind study with subjects with type 2 diabetes, oral semaglutide significantly reduced total daily energy intake by 38.9% compared to placebo, particularly enhancing satiety and appetite control after a fat-rich breakfast [136]. This reduction in energy intake led to a greater decrease in body weight due to a loss in body fat mass and improved eating control.

In a study with mice with obesity, cotadutide was administered following a control or high-fat diet for ten weeks, and treatment was continued for four additional weeks, with hypothalamic arcuate neurons labeled by immunofluorescence and protein expressions assessed using Western blotting. The results showed that cotadutide enhanced POMC and cocaine- and amphetamine-regulated transcript (CART) neuropeptides while reducing neuropeptide Y (NPY) and agouti-related peptide (AgRP), leading to a decrease in appetite [137]. A study conducted on mice aimed to investigate the involvement of caudal brainstem cholecystokinin-expressing neurons in the effect of the GLP-1RA exendin-4. It was found that cholecystokinin-expressing neurons in the caudal brainstem are required for the anorectic and body weight-lowering effects of GLP-1RAs and for the induction of GLP-1RA-induced conditioned taste avoidance [138].

### 4.3. Physiological Pathways That Impact GLP-1 Activity

#### 4.3.1. TGR5 and Its Physiologic Effects Relevant to GLP-1 and Glycogen Metabolism

Takeda G protein-coupled receptor 5 (TGR5), a G protein-coupled bile acid receptor, contributes to improved hepatic insulin sensitivity and subsequent energy metabolism and homeostasis [139]. TGR5 activation leads to downstream GLP-1 secretion [107], ameliorating glucose regulation and glycogen synthesis in animal models with diabetes. TGR5 inhibition of glycogenolysis in hepatic tissue is dependent on GLP-1 activity, as coadministration of a TGR5 agonist and GLP-1 antagonist abolished the suppression of hepatic glycogenolysis [140]. Gq and Gs signaling through fatty acid receptors and TGR5 is necessary for the physiological release of GLP-1. Understanding the implications of upstream GLP-1 signaling may provide context for metabolic disease states, where abnormalities in the TGR5 signaling pathway attenuate robust GLP-1 activity.

#### 4.3.2. PASK Signaling Relevant to GLP-1 and Glycogen Metabolism

PAS kinase (PASK) has been shown to play a pivotal role in glycogen storage, acting as a glucose sensor [141]. In fasted states, the GLP-1 agonist Exendin-4 attenuates glucose-6-phosphatase and maintains glycogen synthase (GYS) activity but has no effect in PASK-deficient mice, whereas they already maintain an elevated level of expression of GYS [142]. PASK-deficient mice have lower expression of AMPK levels, leading to less AMPK phosphorylation and subsequent deactivation of glycogen synthase [143]. GLP-1 agonist Exendin-4 upregulates some genes when PASK is inactivated, such as nuclear factor erythroid 2-related factor 2 (NRF2) and copper–zinc superoxide dismutase (SOD), and others independent of PASK status, such as peroxisome proliferator-activated receptor gamma coactivator 1-alpha (PPargc1a) [144]. As NRF2 and copper–zinc SOD are implicated in mitigating cellular stress (21), and increased cellular oxidation reduces glycogen synthesis [145], the effects of GLP-1As on increasing glycogen synthesis can be further attributed to their ability to attenuate oxidative stress through upregulation of pro-antioxidant genes.

#### 4.3.3. Microbiome Relevance to GLP-1 Activity

The microbiome is necessary for postprandial increases in GLP-1 secretion, as depletion of the microbiome with antibiotic treatment attenuates the postprandial release of GLP-1 [146]. The activity of sodium-glucose-co-transporter-2 (SGLT2) inhibitors induces a change in microbiome diversity, leading to increased GLP-1 production from bacterial-produced post-biotics, noticeably those of tryptophan metabolism [147]. Probiotic administration increased GLP-1 production through the induction of proglucagon and proconvertase activity [148]. Elucidating specific strains, such as *Akkermansia muciniphila*, in the role of increasing endogenous GLP-1 activity and UCP1 expression in brown/beige adipose tissue is vital in understanding how gut health leads to disease states. Obesity-resistant mouse lines show increased brown adipose tissue thermogenesis and GLP-1 production. Fecal transplants from these obesity-resistant lines to high-fat diet (HFD) mouse models attenuate some of the diet-related adiposity and glucose dysregulation, further implicating the gut microbiome in maintaining healthy glucose and energy homeostasis [149].

## 5. Summary

A review of the current literature suggests that many different organ systems either directly or indirectly respond to GLP-1 signaling in relation to glycogen metabolism. The pancreas and central nervous system, specifically hypothalamic structures, have been shown to robustly and directly respond to GLP-1R agonism, mediating anti-diabetic and anti-obesogenic physiology and behaviors, respectively. Both the liver and skeletal muscle have the greatest propensity for glucose uptake and glycogen storage, where GLP-1 signaling is seen to increase glycogen synthesis in these tissues. Future research investigating if glycogen synthesis and storage is increased in these tissues after the pharmacologic use of GLP-1 agonists may be useful in determining if these effects are clinically significant. Furthermore, there remains debate as to whether the liver can directly respond to GLP-1 or if the increases in liver glycogen metabolism are due to GLP-1 augmentation of pancreatic beta-cell insulin secretion. GLP-1 effects on glycogen metabolism in both the liver and skeletal muscle are complex, involving many signaling pathways such as the AKT and MAPK pathways, where many of these pathways and interactions are poorly elucidated and continue to be investigated.

The findings of this review not only found the effects of GLP-1 on glycogen metabolism relevant but also the effects of GLP-1 on downstream adipose tissue thermogenesis as a potential contributing factor towards decreases in body weight seen in individuals prescribed GLP-1 medications. This mechanism remains controversial in explaining the physiologic decreases in weight loss seen in patients [150]. While the increases in thermogenesis following GLP-1RA administration are promising in cell line and animal studies, further studies are needed in humans to determine if this effect is meaningful in contributing to weight loss due to conflicting results of pharmacologic GLP-1RA treatment and energy expenditure in human studies [151].

It is important to also consider the potential effects of GLP-1RA administration on psychiatric conditions. In the context of food-seeking behaviors, GLP-1 administration has been shown to decrease mesolimbic dopaminergic signaling [152]. As mesolimbic dopaminergic pathways are implicated in motivation, perceived energy, and reward-seeking behavior, underactivation of this pathway, potentially through GLP-1RA administration, has been implicated in psychiatric disorders such as depression [153]. However, the effects of GLP-1 on dopamine phenotypes are inconclusive, as a previous study showed Exn-4 administration decreased dopaminergic signaling through increased dopamine transporter (DAT) in rats but not in humans [154]. A recent cohort study demonstrated a significantly increased risk in psychiatric outcomes in patients prescribed GLP-1RA [155], while another study conducted on older adults with T2DM found a decreased risk of depression following GLP-1RA administration [156]. Differences in inclusion criteria, patient demographics, and study design could account for these discrepancies, yet nonetheless evidence exists that GLP-1 activity likely influences mesolimbic signaling through a complex and not yet fully understood mechanism. Individual responses to GLP-1RA could be influenced by specific genotypes, for example, individuals with the Taq1A dopamine receptor 2 polymorphism exhibit decreased binding to dopamine [157]. GLP-1 agonism has been attributed to maintaining dopamine homeostasis, but current research suggests GLP-1 agonism more accurately reduces dopamine signaling in the mesolimbic system regardless of the current state of the system [158]. Previous studies have shown that the Taq1A polymorphism is linked to obesity-related outcomes in children, where individuals with polymorphisms that have decreased basal dopaminergic signaling could potentially be at risk for increased psychiatric side effects following GLP-1RA administration [159]. Consequently, individuals in a state of decreased basal dopamine signaling, known as a hypodopaminergic state, may possess a greater risk for post-GLP-1RA treatment-associated psychiatric conditions such as depression and suicide, where polymorphisms in the DRD2 receptor are likely implicated in this pathophysiology [160,161]. While this is not a comprehensive review of genetic predispositions for potential psychiatric side effects following GLP-1 signaling, it highlights the importance of future research to better understand individual factors that could contribute to conditions such as depression, anxiety, and suicidal ideation following GLP-1 use.

The mechanisms of GLP-1 discussed in this review must be considered through its potential therapeutic effects on conditions related to dysregulated glycogen metabolism, namely sarcopenia. The prevalence of comorbid diagnoses of sarcopenia and obesity has substantially risen over recent years, where many individuals with obesity under the age of 65 now have sarcopenic levels of muscle mass [162]. Clinicians and researchers should address the high rates of skeletal muscle loss following GLP-1RA treatment and attempt to mitigate such losses to prevent complications associated with sarcopenia [163,164]. To date, no research study has attempted to evaluate and quantify muscle or liver glycogen content following GLP-1 administration or the long-term effects of these potential changes on health and related conditions. Previous research has demonstrated increased amino acid catabolism in skeletal muscle with low glycogen content, and further research is needed to determine if skeletal muscle loss in patients taking GLP-1RA can be mitigated through synergistic pharmaceutical or dietary interventions to maximize skeletal muscle glycogen content [165]. Skeletal muscle loss is seen in individuals prescribed GLP-1Ras, and current research aims to attenuate such losses to improve long-term health and outcomes related to muscle wasting and sarcopenia [166]. A preclinical model shows promise in mitigating muscle loss associated with GLP-1RA through the concurrent use of biologics that attenuate muscle-wasting pathways in skeletal muscle [167]. As patients with both obesity and T2DM have dysregulated skeletal muscle glucose uptake and glycogen metabolism compared to individuals with obesity alone, further research should identify and determine if there are differences in losses of skeletal muscle mass among these groups following GLP-1RA treatment. By understanding the interplay of comorbid diagnoses on both the immediate and long-term effects of GLP-1RA treatment, clinicians can use precision medicine in treatment modalities to reduce risks and maximize the therapeutic potential of these pharmacologic agents.

Elucidating the interplay between increased glycogen, decreased energy consumption, and increased energy expenditure may be beneficial in better understanding exactly how GLP-1 agonists exert their anti-obesogenic and anti-diabetic effects. Direct comparisons of the novel GLP-1 agonists on their ability to increase glycogen storage and energy expenditure may provide future therapeutic guidelines in helping physicians treat their patients. Better understanding how GLP-1 directly mediates glycogen metabolism in vivo could assist in understanding dietary influences, where GLP-1 has been found to improve mice pancreatic beta-cell function during a high-fat diet [168]. Due to the widespread use of GLP-1R agonists to treat metabolic dysfunction, obesity, and T2DM, it remains important to understand the systemic physiology of GLP-1R activity. Future research that elucidates the physiological effects of GLP-1 on glycogen metabolism and energy expenditure in humans would explain currently underinvestigated mechanisms that are likely to contribute to the clinical effects of these medications.

## Abbreviations

The following abbreviations are used in this manuscript:

GLP-1	Glucagon-like peptide 1
GLP-1R	Glucagon-like peptide 1 receptor
GLP-1RA	Glucagon-like peptide 1 receptor agonist
GIP	Gastric inhibitory polypeptide
GIP-R	Gastric inhibitory polypeptide receptor
T2DM	Type 2 diabetes mellitus
BMI	Body mass index
DNA	Deoxyribonucleic acid
AKT	Protein kinase B
AMPK	AMP-activated protein kinase
PKA	Protein kinase A
PKC	Protein kinase C
BAT	Brown adipose tissue
PASK	PAS kinase
UCP1	Uncoupling protein 1
TGR5	Takeda G protein-coupled receptor 5
GLUT-4	Glucose transporter 4
TNF-α	Tumor necrosis factor-alpha
GSK-3	Glycogen synthase kinase-3
IL-6	Interleukin-6
G6P	Glucose 6-phosphate
iNKT	Invariant natural killer T-cell
NTS	Nucleus tractus solitarius
UDP-ase	UDP-glucose pyrophosphorylase
PP-1	Protein phosphatase-1
PI3K	Phosphoinositide 3-Kinase
cAMP	Cyclic adenosine monophosphate
LSG	Laparoscopic sleeve gastrectomy
ARC	Arcuate nucleus
Exn-4	Exendin-4
PYY	Peptide YY
IFN	Intestinofugal neuron
HFD	High-fat diet
POMC	Pro-opiomelanocortin
CART	Cocaine- and amphetamine-regulated transcript
NPY	Neuropeptide Y
AgRP	Agouti-related peptide
GYS	Glycogen synthase
NRF2	Nuclear factor erythroid 2-related factor 2
SGLT2	Sodium-glucose-co-transporter-2
SOD	Copper–zinc superoxide dismutase
PPargc1a	Peroxisome proliferator-activated receptor gamma coactivator 1-alpha
NASH	Non-alcoholic hepatic steatosis
CKD	Chronic kidney disease
GFR	Glomerular filtration rate
